# Non-coding RNAs modulate autophagy in myocardial ischemia-reperfusion injury: a systematic review

**DOI:** 10.1186/s13019-021-01524-9

**Published:** 2021-05-22

**Authors:** Fuwen Huang, Jingting Mai, Jingwei Chen, Yinying He, Xiaojun Chen

**Affiliations:** 1 The Fifth People’s Hospital of Zhuhai, Zhuhai City, Guangdong Province China; 2 Sun Yat-Sen Memorial Hospital, Sun Yat-sen University, Guangzhou City, Guangdong Province China; 3 Foshan Hospital of Traditional Chinese Medicine, No.6 Qinren Road, Foshan City, Guangdong Province 528000 PR China

**Keywords:** Non-coding RNAs (ncRNAs), Myocardial ischemia-reperfusion-injury, Autophagy, Molecular mechanisms

## Abstract

The myocardial infarction is the main cause of morbidity and mortality in cardiovascular diseases around the world. Although the timely and complete reperfusion via Percutaneous Coronary Intervention (PCI) or thrombolysis have distinctly decreased the mortality of myocardial infarction, reperfusion itself may lead to supererogatory irreversible myocardial injury and heart function disorders, namely ischemia-reperfusion (I/R) injury. Extensive studies have indicated that non-coding RNAs (ncRNAs), including microRNAs (miRNAs), long noncoding RNAs (lncRNAs) and circular RNAs (circRNAs), play important roles in the progress of myocardial I/R injury, which is closely correlative with cardiomyocytes autophagy. Moreover, autophagy plays an important role in maintaining homeostasis and protecting cells in the myocardial ischemia reperfusion and cardiomyocyte hypoxia-reoxygenation (H/R) progress. In this review, we first introduced the biogenesis and functions of ncRNAs, and subsequently summarized the roles and relevant molecular mechanisms of ncRNAs regulating autophagy in myocardial I/R injury. We hope that this review in addition to develop a better understanding of the physiological and pathological roles of ncRNAs, can also lay a foundation for the therapies of myocardial I/R injury, and even for other related cardiovascular diseases.

## Background

Although advances are made in all aspects of the cardiovascular disease (CVD), it remains one of the most dominating causes of disability and death globally [1]. Relatively, it is reported that there will be almost 23.6 million people dying from CVD by 2030 [2, 3]. More specifically, myocardial infarction (MI) is a primary component in CVD, which can be treated with timely and complete reperfusion with main percutaneous coronary intervention (PCI) and thrombolysis in clinic [4, 5]. The rapid recovery of ischemic myocardial blood flow can prevent myocardial cell death, restrict infarct size, and decrease disability and death, which prominently improves the quality of life of patients with MI. Nonetheless, reperfusion itself may contribute to supererogatory irreversible myocardial injury and/or heart function disorders, namely myocardial I/R injury [6]. Its detrimental effects can result in a series of adverseness and damages such as re-infarction, malignant arrhythmias and heart failure that threaten human health seriously [7]. Currently, myocardial I/R injury is the crucial pathogenesis of CVD that is implicated in several pathological processes, including cell death programs of cardiomyocytes (apoptosis, autophagy and necrosis), inflammation, oxidative stress and mitochondrial dysfunction, contributing to the beginning and progression of myocardial I/R injury [8].

Autophagy is an evolutionarily conserved and important progress in eukaryotes, which is responsible for the turnover of intracellular substances. In this progress, some damaged proteins or organelles are encapsulated by autophagic vesicles with double membrane structure, then sent to lysosomes (animals) or vacuoles (yeasts and plants) for degradation and recycling [9]. The level of autophagy is involved in myocardial I/R injury and cardiomyocyte H/R, which is required for the maintenance of cardiomyocytes homeostasis and protection of cells [10]. Autophagy can also be used as an effective physiological adaptive cause of cell aging and anti I/R-related arrhythmias [11]. Autophagy regulates myocardial I/R injury with the safeguard of cell death at the ischemia period and the inhibition of cell death at the reperfusion period. The mammalian target of rapamycin (mTOR) and Beclin-1 are associated with autophagy-associated signaling pathways, in which mTOR promotes by adenosine 5′-monophosphate (AMP)-activated protein kinase (AMPK) and phosphoinositide 3 kinase (PI3K)/Akt signaling pathway, and Beclin-1 is upregulated during reperfusion [12].

Tremendous research in the past decades have indicated that ncRNAs play an essential role in both the cardiogenesis [13] and cardiac disease, such as acute MI [14, 15], heart failure [16] and hypertrophy [17]. Less than 2% of the human genome contain coding sequences, whereas some of the remaining of genome are transcribed as ncRNAs, including miRNAs, lncRNAs and circRNAs [18]. MiRNAs are a type of single-stranded ncRNAs with 18–24 nucleotides in length that regulate gene expression through inhibition of mRNA translation or degradation and suppression of gene translation [19, 20]. LncRNAs are a group of ncRNAs with 200–100,000 nucleotides in length that modulate gene expression in a post-transcriptional, translational, and epigenetic manner both in pathological and physiological conditions [21, 22]. CircRNAs are a class of ncRNAs with covalently end-to-end connection that function as miRNA sponges, translation modulators, and biomarkers in a wide range of biological processes [23, 24]. It is established that majority of ncRNAs are prominently dysregulated in the heart, which demonstrated that they implicate with the mechanism and therapy of myocardial I/R injury [25]. In this present review, we briefly introduced the biogenesis and functions of ncRNAs, and summarized the role and molecular mechanism of ncRNAs modulating autophagy in myocardial I/R injury.

## The biogenesis and function of ncRNAs

### The biogenesis and function of miRNAs

MiRNAs are generally transcribed into a primary transcript called pri-miRNAs through RNA polymerase II [26] or unusually RNA polymerase III [27], which comprise a 5 ‘terminal cap and a 3 ‘poly-A tail [28]. Subsequently, pri-miRNAs are treated with a complex composed of RNase III enzyme Drosha, double stranded RNA binding protein and DGCR8 (Di George Syndrome Critical Region Gene 8) in the nucleus to obtain premiRNAs with a length of about 70 nucleotides, which fold into a styloid loop structure [29]. Then, premiRNAs are exported to the cytoplasm via the karyophorin Exportin 5 (Exp5) transporter [30]. Once in the cytoplasm, premiRNAs are processed by RNase III enzyme Dicer to produce double stranded RNA with a length of approximately 22 nucleotides. Dicer also initiates the formation of RNA induced silent complex (RISC), which can mediate miRNA expression and gene silencing caused by RNA interference [31]. MiRNAs can silence gene expression through translation inhibition or degradation.

### The biogenesis and function of lncRNAs

LncRNAs are usually generated by RNA polymerase II, which contain 5′ end caps and 3′ poly-A tails similar to their protein-encoding mRNAs [32]. According to their position in the genome relative to protein coding genes, lncRNAs are currently divided into following five types: sense lncRNAs, antisense lncRNAs, bidirectional lncRNAs, intronic lncRNAs and the long intergenic ncRNAs (lincRNAs) [33]. LncRNAs are initially considered as the ‘noise’ or ‘junk’ of genome transcription and a by-product of RNA polymerase II transcription that has no biological function. However, recent studies have shown that lncRNAs are widely involved in many important regulatory processes, such as chromosome silencing, genomic imprinting, chromatin modification, transcriptional activation, transcriptional interference, and nuclear transport [34]. LncRNAs can modulate transcriptional silencing, activate protein coding genes, bind to proteins to mediate their functions, associate with mRNAs to affect their translation, and inhibit the function of miRNAs as competitive endogenous RNAs [35–37].

### The biogenesis and function of circRNAs

During mRNA formation, premRNAs are spliced to wipe off introns and connect exons to produce mature mRNAs [38]. Specifically, plentiful premRNAs can be processed by back-splicing, in which the downstream 5 ‘splice site is connected with the upstream 3’ splice site in reverse order on one or more exons to form circRNAs. On the other hand, the excised intron lariats in the conventional splicing process can occasionally break away from the branch and persist the circular form with 2 ‘, 5’ - phosphodiester bond between the splicing donor and the branch point. These RNA loops are called circular intron RNAs [39]. Therefore, circRNAs are generally grouped into three major types, including circular intron RNAs (ciRNAs), exon intron circRNAs (eiciRNAs) and exon circRNAs (ecircRNAs) [40]. Therein, ciRNAs populated in nucleus are always thought as the coproducts of canonical splicing and back-splicing [39]. EcircRNAs primarily resided in cytoplasm are yielded by back-splicing and act as manifold functions [40]. EiciRNAs localized in nucleus are regarded as intermediate outcome in the generation of ecircRNAs [41]. circRNAs play multiple roles in the biological processes. For instance, both eiciRNAs and ciRNA can impact parental genes [42, 43]. Also, circRNAs can act as miRNA sponges, biomarkers and translation modulators [40].

## NcRNAs regulate autophagy in myocardial I/R-injury

### MiRNAs regulate autophagy in myocardial I/R-injury

MiRNAs are involved in various biological processes that are implicated with cell fate, proliferation, stress response and death [44, 45]. Intriguingly, extensive studies have indicated that miRNAs contribute to myocardial I/R-injury through autophagy. MiR-204 was lowly expressed in myocardial I/R-injury rat model constructed through 30 min ischemia followed by 2 h reperfusion. Furthermore, the expression of microtubule-associated protein 1 light chain 3 (LC3)-II was increased in myocardial I/R-injury rat model, which could be attenuated by miR-204 mimic, suggesting the role of miR-204 in myocardial I/R-injury through autophagy [46]. Another study showed that the expression of miR-204 was also significantly reduced after H9C2 cells were treated with hypoxia for 12 h followed by reoxygenation for another 24 h. The autophagy level was significantly increased with H/R treatment, as evidenced by the elevation of Beclin-1 and the transform of LC3-I to LC3-II, while this effect was reversed after the expression of miR-204 mimics. Mechanistically, the overexpression of SIRT1, a direct target of miR-204, could rescue the declined Beclin-1 level and LC3-II/LC3-I ratio induced by miR-204 overexpression, which was dampened by an autophagy inhibitor, 3-MA. The results demonstrated that miR-204 could weaken the H/R injury via modulating SIRT1-mediated autophagy [47]. It was reported that miRNA-30e was lowly expressed in patients with myocardial I/R-injury as well. Interference of miRNA-30e prominently enhanced the level of LC3B-II, Beclin-1 and p62 in H9C2 cells. Moreover, downregulation of miRNA-30e markedly repressed apoptosis (including decrease of cellular apoptosis, and reduction of the expression of Bax and caspase-3), and the level of iNOS and oxidative stress, which could be dramatically reversed by the suppression of autophagy after treated with 3-MA. Accordingly, miRNA-30e could protect the heart from myocardial I/R-injury through autophagy as well [48]. After rat hearts were subjected with 50 μmol/L sodium hydrosulfide or 10 nmol/L urocortin 2 to build a myocardial I/R injury model, miRNA array was utilized to analyze the regulations of cardiac miRNA. Therein, miRNA-221 was negatively correlated with autophagy potentials. It could decrease the expression of LC3-II in myocardial I/R injury. In addition, the messenger RNA (mRNA) and protein levels of TP53inp1, Ddit4 and p27 were reduced in myocardial I/R injury model treated with miRNA-221 mimic as well [49]. In myoblast H9c2 and neonatal rat ventricular myocytes treated with H/R, miR-221 inhibited the autophagosome formation, which was implicated with targeting the Ddit4/ mTORC1 pathway and repression of Tp53inp1/p62 complex formation. These findings indicated that miR-221 had a protective role against myocardial I/R injury via autophagy [50]. In addition, the expression of miR-142-3p was reduced both in vitro and in vivo myocardial I/R injury model. Myocardial I/R injury promoted autophagy, as shown by enhanced percent of cells positive for LC3 AVs, which was reversed by miR-142- 3p mimic. Additionally, the effect of miR-142-3p on the level of LC3-II/LC3-I ratio, Beclin-1 and p62 was similar to what was observed with the above-mentioned results. Specifically, downregulation of HMGB1 and Rac1 that were targets of miR-142-3p [51, 52] and modulated autophagy [53, 54] restored miR-142-3p inhibitor-enhanced autophagy [55]. Exosome-carried miR-30a inhibitor in the myocardial I/R-injury rat model constructed by joint of the left anterior descending coronary artery observably reduced the protein expression of ULK1 and Beclin-1 in heart tissues compared to that in the myocardial I/R-injury rat model, which demonstrated that miR-30a could inhibit the myocardial I/R-injury via modulating autophagy [56].

Besides, miR-30a was also reported to function in the ischemic postconditioning, which was an endogenous protective mechanism to diminish I/R injury. Upregulation of miR-30a played a cardioprotective role of hypoxia postconditioning in aged cardiomyocytes via repression of BECN1-mediated autophagy, which could be abolished by downregulation of miR-30a. Mechanistically, the level of DNA hypomethylation mediated by DNA methyltransferase 3b at miR-30a promoter was declined with hypoxia postconditioning treatment, thereby resulting in overexpression of miR-30a [57]. Another experiment showed that the expression of miR181a-1, miR139-3p and Beclin-1 was reduced in myocardial I/R-injury model, which could be rescued with postconditioning treatment, indicating the role of miR181a-1 and miR139-3p in myocardial I/R-injury model via regulating autophagy [58].

H/R is one of principal components of myocardial I/R-injury, and the level of miRNAs is fleetly interfered when cardiomyocytes are subjected with H/R [59]. miR-325 was highly expressed in anoxia/reoxygenation (A/R) and I/R injury. Autophagy was potentiated by overexpression of the miR-325, while attenuated by downregulation of miR-325. Mechanistically, the E2F1/miR-325/ARC signaling axis that modulated autophagy was implicated with myocardial I/R-injury [60]. Shao et al. used Langendorff perfusion to build an I/R model in rats, and dealt neonatal rat cardiomyocytes with H/R to construct an in vivo model. Overexpression of miR-34a mimics declined the level of LC3-II, p62 and autophagy after H/R injury, which suggested that miR-34a could suppress the level of autophagy after I/R, thus diminishing myocardial I/R injury [61]. miR-429 was signally down-regulated both in MI hearts and AR-induced cardiomyocytes. miR-429 overexpression showed a decrease in the number of GFP-LC3B labelled cells, vesicle and autophagosome in every cardiomyocyte, whereas suppression of miR-429 inverted the above-mentioned effect. Additionally, the level of LC3BI/II, p62 and ATG13 was memorably enhanced when inhibition of miR-429 both in vivo and in vitro. Importantly, the MO25/LKB1/AMPK signal pathway mediated autophagy was associated with the role of miR-429 in myocardial A/R injury [62]. Similarly, the expression of miR-497 was also observably reduced both in MI hearts and cultured neonatal rat cardiomyocytes. Disturbance of miR-497 augmented autophagic flux, and both in vivo and in vitro study showed that LC3B-II level was reduced by upregulation of miR-497 and increased by downregulation of miR-497, respectively. These results suggested that repression of miR-497 alleviated myocardial A/R injury through improving autophagy [63]. It was also exhibited that miR-638 was down-regulated in the human cardiomyocytes treated with H/R. Also, autophagy was improved with H/R treatment, which could be attenuated by the transfection of miR-638 mimic. Moreover, miR-638 could target the 3′-untranslated region of ATG5 to inhibit the ATG5 level. Therefore, upregulation of miR-638 ameliorated the H/R-induced autophagy via targeting ATG5 [64]. Another study indicated that miR-431 was lowly expressed in human cardiomyocytes treated with H/R. H/R treatments strengthened the autophagy level, which was partly rescued by the transfection of miR-431 mimic. Consistently, miR-431 reduced the ATG3 expression through targeting the 3′-untranslated region of ATG3. Thus, upregulation of miR-431 mitigated H/R through ATG3 [65]. After exosomes obtained from bone marrow mesenchymal stem cells (MSCs) included an elevated level of miR-29c were treated with H/R, their protective efficacy was distinctly decreased, which was associated with the level of exosomal miR-29c. Moreover, miR-29c could target the PTEN/AKT/mTOR signal pathway to reduce superabundant autophagy, thereby protecting heart from I/R injury [66].

MiRNAs played an important role in the therapy for myocardial I/R-injury through autophagy. Autophagy-related genes (including Beclin-1, Atg5, Atg7 and Atg12) were prominently lowly expressed in miRNAs let-7b-transfected MSCs. Moreover, let-7b-transfected MSCs injected into myocardium notably improved left ventricular function and microvessel density. This means that let-7b could protect MSCs injected into myocardium from autophagy, raising the efficacy of MSCs therapy [67]. A previous study revealed that miR-30a was lowly expressed in myocardial I/R cells, which could be rescued by salvianolic acid B in a dose-dependent manner. Moreover, salvianolic acid B inhibited autophagy, which promoted for cell survival in myocardial I/R cells. More importantly, miR-30a inhibitor inverted the anti-autophagy effect of salvianolic acid B against I/R injury. Mechanistically, PI3K/Akt signaling axis was involved in the protective role of salvianolic acid B in miR-30a-mediated autophagy, as evidenced by PI3K inhibitor LY294002 abolished the effect [68]. Another in vitro and in vivo study showed that myocardial I/R injury enhanced autophagosomes, thus augmenting autophagic flux, which was dampened by pretreatment with epigallocatechin gallate. Furthermore, in vitro study revealed that epigallocatechin gallate rescued the downregulation of miR-384 targeting to Beclin-1. Both upregulation of miR-384 and downregulation of Beclin-1 prominently autophagy induced by I/R injury, concurring with the activation of PI3K/Akt pathway [69]. Rosuvastatin boosted the levels of miR-17-3p and LC3II/LC3I in myocardial I/R cells. Knockdown of miR-17-3p decreased LC3II/LC3I level, while overexpression of miR-17-3p enhanced LC3II/LC3I level. These results indicated that autophagy occurred by upregulating the level of miR-17-3p [70]. Table 1 showed the list and targets/pathways of miRNAs in myocardial I/R injury.

**Table 1:** List and targets/pathways of miRNAs in myocardial I/R injury

miRNA	Change in expression	Downstream targets/pathways	Study
miR-204	downregulation	increase of LC3-II expression	[45, 46]
miR-30e	downregulation	increase of LC3B-II, Beclin-1 and p62 level	[47]
miR-221	upregulation	decrease of LC3-II expression	[48, 49]
miR-142-3p	downregulation	increase of percent of cells positive for LC3 Avs, and the level of LC3-II/LC3-I ratio, Beclin-1 and p62	[54]
miR-30a	downregulation	decrease of ULK1 and Beclin-1, and mediated by PI3K/Akt signaling axis	[55, 56, 67]
miR181a-1	downregulation	decrease of Beclin-1 expression	[57]
miR139-3p	downregulation	decrease of Beclin-1 expression	[57]
miR-325	upregulation	A E2F1/miR-325/ARC signaling axis modulating autophagy	[59]
miR-34a	downregulation	decrease of LC3-II, p62 and autophagy level	[60]
miR-429	upregulation	decrease in the number of GFP-LC3B labelled cells, vesicle and autophagosome, increase of the level of LC3BI/II, p62 and ATG13	[61]
miR-497	downregulation	decrease of autophagic flux, and increase of LC3B-II level	[62]
miR-638	downregulation	increase of autophagy via targeting ATG5	[63]
miR-431	downregulation	decrease of ATG3 expression	[64]
miR-29c	upregulation	decrease of autophagy by targeting the PTEN/AKT/mTOR signal pathway	[65]
let-7b	downregulation	decrease of Beclin-1, Atg5, Atg7 and Atg12 expression	[66]
miR-384	downregulation	increase of autophagosomes and autophagic flux, the activation of PI3K/Akt pathway	[68]
miR-17-3p	upregulation	increase of LC3II/LC3I level	[69]

### LncRNAs modulated autophagy in myocardial I/R-injury

Similarly, lncRNAs could be involved in the heart [71] and also regulate autophagy in myocardial I/R injury. Yu et al. revealed that lncRNA MALAT1 enhanced cardiomyocyte autophagy by negatively modulating the expression of miR-204 [72]. Since miR-204 functioned in modulating autophagy via LC3-II during myocardial I/R injury [46], a MALAT1/miR-204/LC3-II signaling axis was speculated to regulate autophagy in myocardial I/R injury [73]. LncRNA TUG1 was highly expressed in myocardial I/R injury both in vitro and in vivo. Downregulation of tautine upregulated gene 1 (TUG1) by siRNA significantly suppressed autophagy, as detected by percent of cells containing LC3+ AVs, and the expression of LC3-I, LC3-II, Beclin-1 and p62. Functionally, TUG1 sponged miR-142-3p and alleviated myocardial I/R injury through targeting HMGB1- and Rac1-induced autophagy [55]. LncRNA PVT1 was upregulated in human AC16 cardiomyocytes challenged with H/R treatment. Interference of PVT1 expression alleviated autophagy, as determined by the decreased expression levels of LC3-II and Beclin-1, the increased expression of p62, and the reduced accumulation of autophagic vacuoles. Moreover, PVT1 could sponge miR-186 that directly targeted with the 3′-UTR of human Beclin-1 mRNA. Thus, miR-186 inhibitor declined the effects of PVT1 downregulation on autophagy as detected and described above [74]. Myocardin, a nuclear protein was highly expressed during I/R-injury, and its downregulation repressed autophagy and diminished myocardial infarction. P53, a tumor suppressor protein and always acting as a transcription factor, modulated cardiomyocytes autophagy and myocardial I/R injury through modulating the myocardin expression. LncRNA CAIF (cardiac autophagy inhibitory factor) could directly bind to p53 and prevent p53-mediated myocardin transcription, which leaded to the reduction of myocardin expression. Totally, CAIF inhibited cardiac autophagy and protected hearts from myocardial infarction via a CAIF-p53-myocardin signaling axis [75]. Besides, lncRNA nuclear-enriched abundant transcript (Neat1) was upregulated in diabetic mice with myocardial I/R injury, which further exacerbated myocardial I/R injury by promoting myocardial autophagy via upregulation of Foxo1 to enhance H/R injury [76].

It was demonstrated that lncRNAs could sponge miRNAs to modulate autophagy in myocardial I/R injury. LncRNA autophagy promoting factor (APF) was indicated to sponge miR-188-3p directly targeting ATG7 to modulate autophagy and myocardial infarction. Downregulation of APF diminished autophagy and cardiac dysfunction through the elevation of miR-188-3p and decline of ATG7. The results revealed that APF prevented MI and heart failure via APF/miR-188-3p/ATG7 signaling axis [77]. Endoplasmic reticulum stress (ERS) is also one of main pathogenesis of myocardial I/R injury and MI. Li et al. [78] used Tunicamycin (Tm) to triger ERS, and found lncRNA discrimination antagonizing non-protein coding RNA (Dancr) was lowly expressed in Tm-induced group. Tm also triggered autophagy, as evidenced by the increase of the level of Beclin 1 and LC3II/I ratio, and the decrease of the expression of p62. Furthermore, overexpression of Dancr promoted autophagy, as indicated by the raise of Beclin 1 and LC3II/I expression, and also prominently downregulated the expression of miR-6324. The directly binding between Dancr and miR-6324 was verified by the dual-luciferase reporter assay. Overexpression of miR-6324 gently rescued the effects of Dancr overexpression on autophagy. These finding indicated that upregulation of lncRNA Dancr sponging miR-6324 prevented myocardial I/R injury, thereby augmenting autophagy and restoring ER homeostasis. LncRNA TTTY15 inhibited autophagy and myocardial I/R injury through targeting miR-374a-5p. Hence, TTTY15 modulated the miR-374a-5p expression, thereby impacting the level of FOXO1 and autophagy in myocardial I/R injury [79]. The global differential expression of lncRNAs analyzed by microarray analysis showed that 797 lncRNAs were differentially expressed in the H/R group. Therein, the repression lncRNA-HRIM via specific siRNAs protected cells from death through diminishing autophagy in H9c2 myocytes during H/R [80]. LncRNA NEAT1 associated with the development of various diseases was upregulated in peripheral blood and mouse cardiomyocytes during MI, which markedly increased the proliferation and migration of cardiomyocytes. It was indicated that NEAT1 suppressed miR-378a-3p level, and miR-378a-3p repressed Atg12 level by target gene prediction and screening, luciferase reporter assays. Additionally, lncRNA NEAT1 sponged miR-378a-3p to modulate expression of Atg12 and related autophagic factors [81]. Additionally, lncRNA AK088388 was demonstrated to directly bind to miR-30a using software analysis and dual-luciferase reporter assays. The expression of miR-30a was decreased, whereas that of AK088388, Beclin-1, and LC3-II was increased in H/R cardiomyocytes. miR-30a suppressed the level of AK088388, Beclin-1, and LC3-II, while AK088388 enhanced the Beclin-1 and LC3-II expression and repressed the expression of miR-30a. All the results suggested that AK088388 competitively join to miR-30a, facilitating the Beclin-1 and LC3-II expression, and autophagy [82]. Long noncoding RNA component of mitochondrial RNA processing endoribonuclease (RMRP) negatively modulated the level of miR-206, and RMRP overexpression exacerbated hypoxia injury through downregulation of miR-206 in H9c2 cells. Moreover, overexpression of miR-206 could invert the effect of RMRP overexpression activating PI3K/AKT/mTOR pathway in hypoxia-induced H9c2 cells. Since the role of miR-206 in hypoxia injury was mediated by targeting ATG3, a RMPR/miR-206/ATG3 axis might be involved in alleviating the myocardial I/R injury via activation of PI3K/Akt/mTOR pathway [83]. Oxygen-glucose deprivation and reoxygenation (OGD/R) treatment enhanced the expression of long non-coding RNA FOXD3 antisense RNA 1 (FOXD3-AS1), which was accelerated the level of LC3 II, Beclin1, ATG5, and reduced the expression of p62. Moreover, overexpression of FOXD3-AS1 activated NF-κB/iNOS/COX2 signaling pathway, which was obstructed by 3 M. These findings revealed that FOXD3-AS1 promoted myocardial I/R injury via enhancing autophagy mediated by NF-κB/iNOS/COX2 axis [84].

Additionally, owing to the aggravated effect on infarct sizes and dysfunction after myocardial I/R injury, diabetes is regarded as a highly risk factor for the poor prognosis. Knockdown of AK139328 dramatically enhanced the miR-204-3p level in diabetic mice with myocardial I/R injury. Furthermore, downregulation of AK139328 and upregulation of miR-204-3p suppressed the level of LC3-I*/*LC3-II, ATG5, ATG7 and p62, thus reducing the H*/*R injury. Collectively, AK139328 directly regulated miR-204-3p and then repressed cardiomyocyte autophagy in diabetes [85]. In addition, it was reported that morphine postconditioning (MpostC) declined myocardial reperfusion injury. Thus, Chen et al. found that MpostC treatments prominently diminished cell autophage, increased the lncRNA UCA1 expression, and decreased the miR-128 level compared to these in I/R cardiac tissues. Moreover, it was demonstrated that the binding of UCA1 and miR-128 using RNA immunoprecipitation (RIP) and RNA pull-down assays, and that of miR-128 and HSP70 using the luciferase reporter assay, which eventually suggested that the UCA1/miR-128/HSP70 signaling axis was involved in the protective effect of MpostC on myocardial I/R injury [86]. Table 2 shows the list and targets/pathways of lncRNAs in myocardial I/R injury.

**Table 2:** List and targets/pathways of lncRNAs in myocardial I/R injury

lncRNA	Change in expression	Downstream targets/pathways	Study
MALAT1	upregulation	a MALAT1/miR-204/LC3-II signaling axis to regulate autophagy	[70, 71]
TUG1	upregulation	increase of percent of cells containing LC3+ AVs, and the expression of LC3-I, LC3-II, Beclin-1 and p62	[54]
PVT1	upregulation	increase of expression levels of LC3-II and Beclin-1, the decrease of expression of p62 and the accumulation of autophagic vacuoles	[72]
CAIF	downregulation	inhibition cardiac autophagy via a CAIF-p53-myocardin signaling axis	[73]
Neat1	upregulation	promoting myocardial autophagy via upregulation of Foxo1	[74, 79]
APF	upregulation	modulating autophagy via APF/miR-188-3p/ATG7 signaling axis	[75]
Dancr	downregulation	increase of the level of Beclin 1 and LC3II/I ratio, and the decrease of the expression of p62	[76]
TTTY15	upregulation	inhibition of autophagythrough targeting miR-374a-5p	[77]
HRIM	upregulation	reduction of autophagy	[78]
AK088388	upregulation	increase of Beclin-1, and LC3-II expression	[80]
RMRP	upregulation	a RMPR/miR-206/ATG3 axis involved in autophagy via activation of PI3K/Akt/mTOR pathway	[81]
FOXD3-AS1	upregulation	increase of level of LC3 II, Beclin1, ATG5, and reduced the expression of p62, mediated by NF-κB/iNOS/COX2 axis	[82]
AK139328	upregulation	increase of the level of LC3-I*/*LC3-II, ATG5, ATG7 and p62	[83]
UCA1	upregulation	reduction of cell autophage	[84]

### CircRNAs modulated autophagy in myocardial I/R-injury

The covalently closed structure of circRNAs gives them high stability that makes them play vital roles in myocardial I/R injury. CircRNA autophagy-related circular RNA (ACR) mediated cardiomyocyte autophagy by directly targeting Dnmt3B and obstructing Dnmt3B-mediated DNA methylation of Pink1 promoter to activate the expression of Pink1, and Pink1 was demonstrated to phosphorylate FAM65B at serine 46 to inhibit autophagy and decrease MI size. All these results suggested that the protective role of the ACR/Pink1/FAM65B axis in the myocardial I/R injury [87]. CircPAN3 was downregulated in myocardial I/R injury, and circPAN3 overexpression markedly repressed autophagy, as further detected by a decrease of autophagic vacuoles. Moreover, circPAN3 directly targeted with miR-421 to regulate myocardial I/R injury, and miR-421 negatively modulate Pink1 (phosphatase and tensin homologue-induced putative kinase 1) through binding sites. Downregulation of Pink1 abrogated antiautophagy induced by circPAN3 overexpression in myocardial I/R injury. Therefore, circPAN3 provided protective role in the myocardial I/R injury via circPAN3-miR-421-Pink1 signaling axis-mediated autophagy [88]. Pretreatment with Salidroside, exhibiting protective effect on cardiovascular system, autophagy was prominently suppressed in myocardial I/R injury rat model, which was gently rescued by rapamycin (RAPA), an autophagic agonist, accompanied with the upregulation of circ-0000064. Thus, the protective role of Salidroside in myocardial I/R injury was associated with the upregulation of circ-0000064 and the repression of autophagy [89].

## Conclusion

**Fig. 1:**
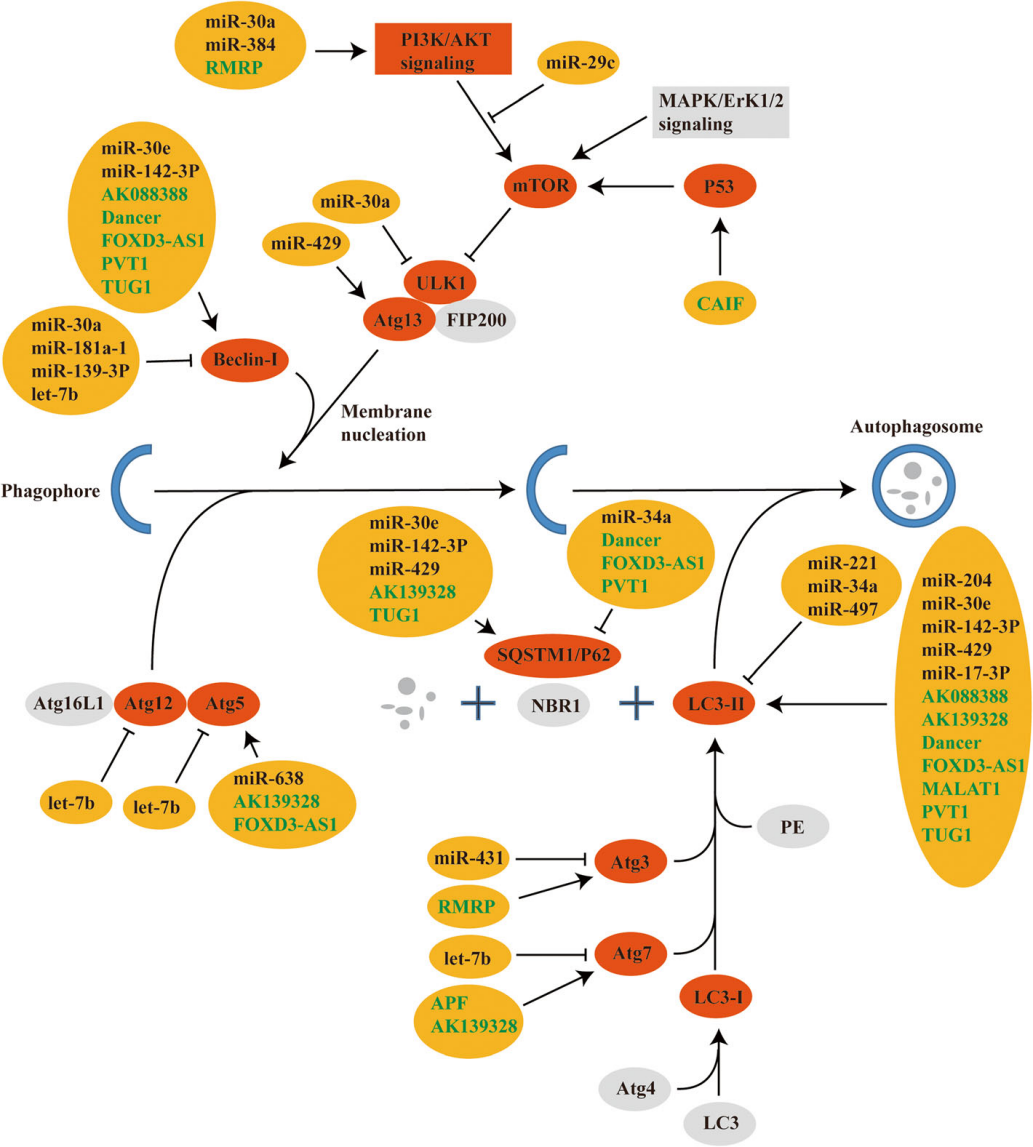
A diagram showing regulation of autophagy pathway by ncRNAs after myocardial ischemia-reperfusion injury. The diagram exhibited the various ncRNAs and their targets that are involved in the modulation of autophagy during myocardial ischemia-reperfusion injury. The listed targets included LC3-II, p62, Beclin-1, ULK1, Atg3, Atg5, Atg7, Atg12, Atg13, PI3K/AKT signaling pathway. MiRNAs show as in black in the yellow box, and lncRNAs in green

Myocardial I/R injury is an inevitable problem in the treatment of ischemia that can lead to re-infarction, malignant arrhythmias and heart failure, thereby threatening human health severely. Several pathogenesis’ pathways are involved in myocardial I/R injury, including autophagy. Autophagy plays various roles both in physiological and pathophysiological processes, and dysregulation of autophagy is relevant in many cardiac diseases, such as ischemic heart disease, dilated cardiomyopathy, and heart failure. In recent years, ncRNAs has been demonstrated to develop essential roles in myocardial I/R injury mediated by autophagy. Therefore, we summarized and discussed the role of ncRNAs in myocardial I/R injury mediated by autophagy (Fig. 1), which is particularly significant for the understanding the pathogenesis and molecular mechanism of myocardial I/R injury, even eventually improving cardiovascular disease. Owing to the dysregulation of ncRNAs in myocardial I/R injury, they are generally considered as biomarkers or therapeutic targets for myocardial I/R injury. Noteworthily, many ncRNAs may correspond with the identical miRNA response element to form competitive endogenous RNAs (ceRNAs), which take part in the progression of myocardial I/R injury. Thus, lncRNAs and circRNAs can sponge miRNAs to generate lncRNAs/circRNAs-miRNA-mRNA signaling axis to regulate myocardial I/R injury, and cardiovascular diseases.

## Data Availability

All data generated or analyzed during this study are included in this published article.

## Abbreviations

I/R injury: Ischemia-reperfusion injury
PCI: Percutaneous Coronary Intervention
ncRNAs: Non-coding RNAs
miRNAs: MicroRNAs
lncRNAs: Long noncoding RNAs
circRNAs: Circular RNAs
H/R: Hypoxia-reoxygenation
CVD: Cardiovascular disease
MI: Myocardial infarction
mTOR: Mammalian target of rapamycin
AMPK: Adenosine 5′-monophosphate (AMP)-activated protein kinase
PI3K: Phosphoinositide 3 kinase
DGCR8: Di George Syndrome Critical Region Gene 8
Exp5: Exportin 5
RISC: RNA induced silent complex
lincRNAs: Long intergenic ncRNAs
ciRNAs: Circular intron RNAs
eiciRNAs: Exon intron circRNAs
ecircRNAs: Exon circRNAs
LC3: Microtubule-associated protein 1 light chain 3
mRNA: Messenger RNA
A/R: Anoxia/reoxygenation
MSCs: Mesenchymal stem cells
TUG1: Tautine upregulated gene 1
CAIF: Cardiac autophagy inhibitory factor
Neat1: Nuclear-enriched abundant transcript
APF: Autophagy promoting factor
ERS: Endoplasmic reticulum stress
Tm: Tunicamycin
Dancr: Discrimination antagonizing non-protein coding RNA
RMRP: RNA component of mitochondrial RNA processing endoribonuclease
OGD/R: Oxygen-glucose deprivation and reoxygenation
AS: Antisense
MpostC: Morphine postconditioning
RIP: RNA immunoprecipitation
ACR: Autophagy-related circular RNA
Pink1: Phosphatase and tensin homologue-induced putative kinase 1
RAPA: Rapamycin
ceRNAs: Competitive endogenous RNAs
